# High-Throughput Screening and Molecular Dynamics Simulation of Natural Product-like Compounds against Alzheimer’s Disease through Multitarget Approach

**DOI:** 10.3390/ph14090937

**Published:** 2021-09-18

**Authors:** Danish Iqbal, Md Tabish Rehman, Abdulaziz Bin Dukhyil, Syed Mohd Danish Rizvi, Mohamed F. Al Ajmi, Bader Mohammed Alshehri, Saeed Banawas, M. Salman Khan, Wael Alturaiki, Mohammed Alsaweed

**Affiliations:** 1 Department of Medical Laboratory Sciences, College of Applied Medical Sciences, Majmaah University, Majmaah 11952, Saudi Arabia; a.dukhyil@mu.edu.sa (A.B.D.); b.alshehri@mu.edu.sa (B.M.A.); s.banawas@mu.edu.sa (S.B.); w.alturaiki@mu.edu.sa (W.A.); m.alsaweed@mu.edu.sa (M.A.); 2 Department of Pharmacognosy, College of Pharmacy, King Saud University, Riyadh 11451, Saudi Arabia; mrehman@ksu.edu.sa (M.T.R.); malajmii@ksu.edu.sa (M.F.A.A.); 3 Department of Pharmaceutics, College of Pharmacy, University of Hail, Hail 81442, Saudi Arabia; sm.danish@uoh.edu.sa; 4 Health and Basic Sciences Research Center, Majmaah University, Majmaah 15341, Saudi Arabia; 5 Department of Biomedical Sciences, Oregon State University, Corvallis, OR 97331, USA; 6 Clinical Biochemistry & Natural Product Research Laboratory, Department of Biosciences, Integral University, Lucknow 226026, India; contactskhan@gmail.com

**Keywords:** Alzheimer’s disease, multitarget, molecular dynamics simulations, natural-like compounds, virtual screening

## Abstract

Alzheimer’s disease (AD) is a progressive neurological disorder that affects 50 million people. Despite this, only two classes of medication have been approved by the FDA. Therefore, we have planned to develop therapeutics by multitarget approach. We have explored the library of 2029 natural product-like compounds for their multi-targeting potential against AD by inhibiting AChE, BChE (cholinergic pathway) MAO-A, and MOA-B (oxidative stress pathway) through in silico high-throughput screening and molecular dynamics simulation. Based on the binding energy of these target enzymes, approximately 189 compounds exhibited a score of less than −10 kcal/mol against all targets. However, none of the control inhibitors exhibited a binding affinity of less than −10 kcal/mol. Among these, the top 10 hits of compounds against all four targets were selected for ADME-T analysis. As a result, only F0850-4777 exhibited an acceptable range of physicochemical properties, drug-likeness, pharmacokinetics, and suitability for BBB permeation with high GI-A and non-toxic effects. The molecular dynamics study confirmed that F0850-4777 remained inside the binding cavity of targets in a stable conformation throughout the simulation and Prime-MM/GBSA study revealed that van der Waals’ energy (ΔG_vdW_) and non-polar solvation or lipophilic energy (ΔG_Sol_Lipo_) contribute favorably towards the formation of a stable protein–ligand complex. Thus, F0850-4777 could be a potential candidate against multiple targets of two pathophysiological pathways of AD and opens the doors for further confirmation through in vitro and in vivo systems.

## 1. Introduction

Neurological disorders including Alzheimer’s disease (AD) have a significant negative impact on the mental, psychological, physical, and economic health of patients and their caregivers [1,2]. Almost 50 million people are affected globally from Alzheimer and other dementias [2]. AD is the second leading cause of death among high-income countries, and the seventh leading cause of death worldwide, ranking sixth in Saudi Arabia (currently over 130,000 cases) [3,4]. Patients with AD exhibit memory loss, agitation, dysphoria, apathy, aberrant motor behavior, problems with speaking or writing, and cognitive impairments [1].

Alzheimer’s disease has a very intricate etiology, and several reports have hypothesized that the four major pathophysiological pathways (oxidative stress, amyloid-beta pathway, tau pathway, and cholinergic pathway) are responsible for the progression of AD [5,6,7,8,9,10,11]. These pathways include the formation of senile plaques through amyloid-beta (Aβ) plaque deposition [8], the agglomeration of neurofibrillary tangles after tau neurofibrillary degeneration [12], the disruption of cholinergic activity [11], and oxidative stress [10,13].

Oxidative stress is promoted by an increased production of hydrogen peroxide through the catalytic action of monoamine oxidases (MAO-A and MAO-B) on the primary amine deamination of major neurotransmitters [13], which results in tissue damage, especially in brain cell and disrupts the blood–brain barrier, which could lead to AD [10]. It has also been well established that during AD, there is deterioration of cholinergic neuron-rich regions, resulting in the decline of acetylcholine (ACh) levels, which are believed to be associated with memory loss, agitation, and apathy [11,14]. Moreover, cholinesterase enzymes such as acetylcholinesterase (AChE) and butyrylcholinesterase (BChE), have been found to further decrease the concentration of acetylcholine (ACh) by hydrolyzing it [8,9].

Until now, the US Food and Drug Administration (FDA) has approved two classes of medications to treat AD: (1) cholinesterase inhibitors (donepezil, galantamine, and rivastigmine) and (2) an NDMA receptor antagonist (memantine). However, although these drugs relieve some symptoms and have beneficial effects on cognition and function, they do not treat neuropsychiatric symptoms and do not slowdown or stop the progression of the disease. Moreover, these medications have several side effects including nausea, vomiting, loss of appetite, headache, constipation, confusion, and dizziness [15,16].

At present, there is a lack of disease-modifying medications or a complete cure for AD. The enzymes that promote the pathways responsible for the progression of AD include AChE, BChE, MAO-A, and MOA-B, which would need to be targeted individually or in combination [5,6,17,18,19,20,21]. Advances in computing have recently allowed for the development of various cheminformatics approaches for the faster screening and optimization of bioactive compounds through enzyme inhibition [22,23,24,25,26,27].

Since nature has an endless resource of bioactive compounds, it would be economical and safe to obtain bioactive moieties to produce novel multitarget agents against Alzheimer’s disease [18,28]. Due to the popularity and therapeutic potential of natural products and their derivatives, Life Chemicals Inc. have recently developed proprietary of synthetic compounds, namely, natural product-like compounds, based on cheminformatics and substructure searches (www.lifechemicals.com). However, a “one disease, one target, one drug” strategy is limited by its inability to completely cure complex diseases, such as neurodegenerative diseases or mood disorders [29,30]. These limitations have driven us to explore the development of therapeutics using multiple targeted approaches aimed at several different pathological cascades of AD simultaneously.

In the present study, we explored a library of natural product-like compounds for their multi-targeting (AChE, BChE, MAO-A, MOA-B) potential against AD through in silico high-throughput screening and ADME-T analysis. Furthermore, the validation of the best hit via molecular dynamics simulation was also conducted. To the best of our knowledge, this study is the first to explore this library of natural product-like compounds for multi-targeting against AD.

## 2. Results and Discussion

### 2.1. Virtual Screening Analysis

Although the process of drug discovery is time-consuming and expensive, new drugs are needed to fulfill unmet clinical needs [31]. The number of drug or lead-like molecules available in different databases is estimated to be as high as 1 × 10^24^. Moreover, the structures of potential drug target molecules are increasingly being added to the Protein Data Bank on a regular basis. Thus, to deal with such a large number of molecules and to assist in the drug discovery process, computer-aided drug discovery techniques, such as in silico virtual screening, play a significant role owing to their faster speed and lower cost compared to in vitro high-throughput screening [32]. In the present study, we employed in silico virtual screening, molecular docking, and molecular dynamics simulation to identify a novel inhibitor against multiple targets of Alzheimer’s disease such as AChE, BChE, MAO-A, and MAO-B.

All natural-like compounds in the library and three control inhibitors (Tacrine, Harmine, and Safinamide) against the target proteins (AChE, BChE, MOA-A, and MOA-B) were subjected to docking analysis, generating 10 binding combinations. Based on the binding energy (ΔG), 189 out of 2029 compounds exhibited binding energy score of −10 to −12.9 kcal/mol, −10 to −12.6 kcal/mol, and −10 to −13.6 kcal/mol against AChE, BChE, and monoaminoxidases, respectively. The control inhibitors, such as Tacrine, exhibited a binding affinity of −8.5 kcal/mol and −8.4 kcal/mol against AChE and BChE, respectively. Harmine got binding affinity of −8.7 kcal/mol for MOA-A and Safinamide showed −9.5 kcal/mol of binding affinity against MOA-B. Among these 189 compounds, the top 10 hits of compounds against all four targets were selected (Table 1) for further analysis.

### 2.2. Prediction of Physicochemical, Pharmacokinetics Properties, Drug-Likeness, and Toxicity Potentials

Natural product-like compounds’ physicochemical properties drug-likeness and pharmacokinetics were evaluated using the SwissADME tool [33]. Among the top 10 hits analyzed against targets of Alzheimer’s disease (AChE, BChE, MOAA, and MOAB), five compounds (F0870-0001, F3293-0320, F3385-6048, F1865-0198, and F3139-1218) were found to be unsuitable for BBB permeation. However, all of the compounds had a molecular mass of less than 500 g/mol, showed high gastrointestinal absorption, and showed zero violation of Lipinski’s rule. Moreover, five other compounds (F1094-0205, F1094-0201, F0850-4777, F1094-0200, and F3139-1101) were found to be suitable for BBB permeation including the acceptable range of other parameters (Table 2). Considering the analyzed physicochemical properties and absorption potential, further toxicological investigation was carried out and found that only three compounds (F0850-4777, F3293-0320, and F3385-6048) exhibited no toxicity for all the tested parameters (Table 3). The results showed that F0850-4777 (3-(2-methoxyphenyl)-4-oxo-4H-chromen-7-yl 4-methylbenzoate) has a higher affinity towards all the target proteins and found acceptable range of physicochemical properties, drug-likeness, and pharmacokinetics (Figure S1). This confirmed its amelioration of Alzheimer’s disease and was selected for molecular docking and molecular dynamics simulation analysis.

### 2.3. Molecular Docking Analysis

**Figure 1:**
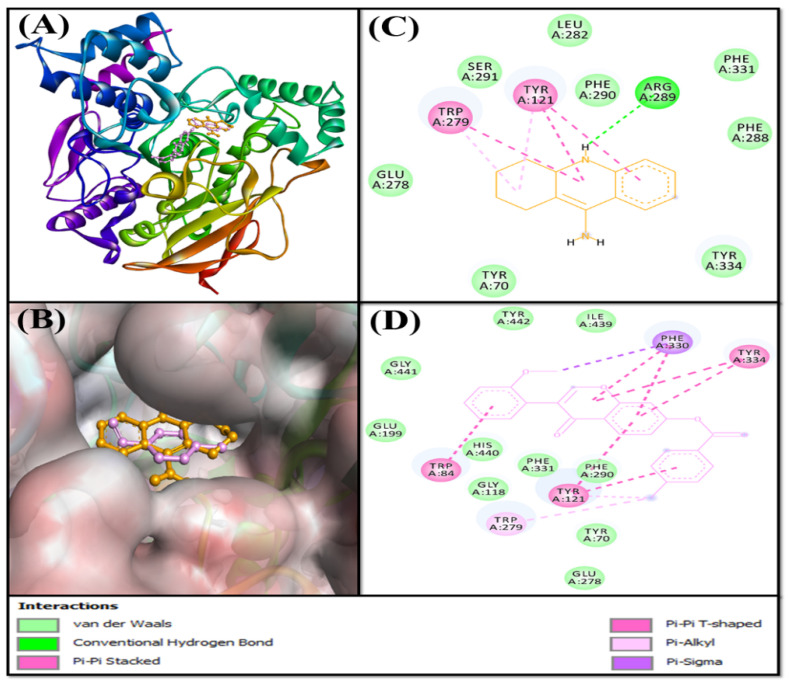
Interaction of target protein, AChE with F0850-4777 and their respective control ligands. (**A**) Position of F0850-4777 and Tacrine in AChE. (**B**) Interactions between AChE and Tacrine. (**C**) Superimposed image of F0850-4777 and Tacrine in AChE. (**D**) Interactions between AChE and F0850-4777.

**Figure 2:**
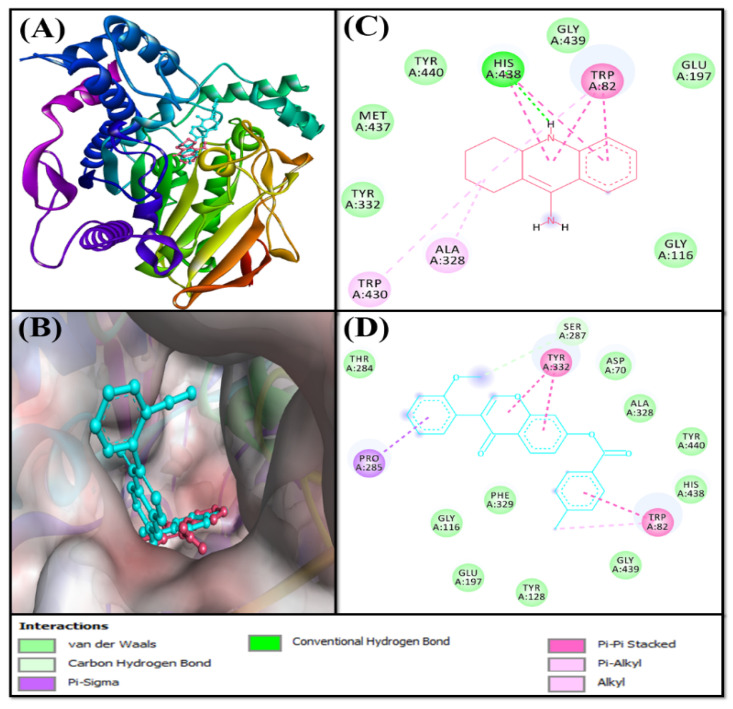
Interaction of target protein, BChE with F0850-4777 and their respective control ligands. (**A**) Position of F0850-4777 and Tacrine in BChE. (**B**) Interactions between BChE and Tacrine (**C**). Superimposed image of F0850-4777 and Tacrine in BChE. (**D**) Interactions between BChE and F0850-4777.

**Figure 3:**
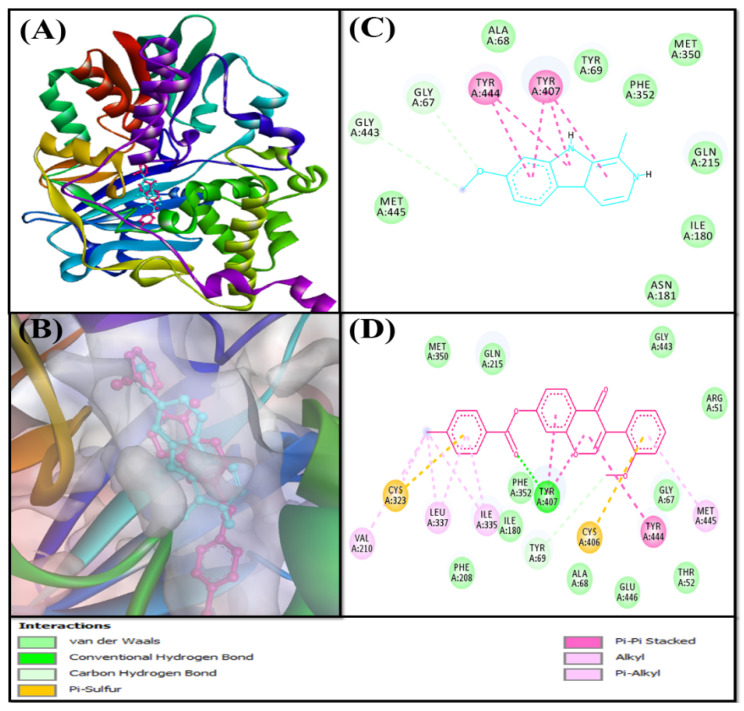
Interaction of target protein, MAO-A with F0850-4777 and their respective control ligands. (**A**) Position of F0850-4777 and Harmine in MAO-A. (**B**) Interactions between MAO-A and Harmine. (**C**) Superimposed image of F0850-4777 and Harmine in MAO-A. (**D**) Interactions between MAO-A and F0850-4777.

**Figure 4:**
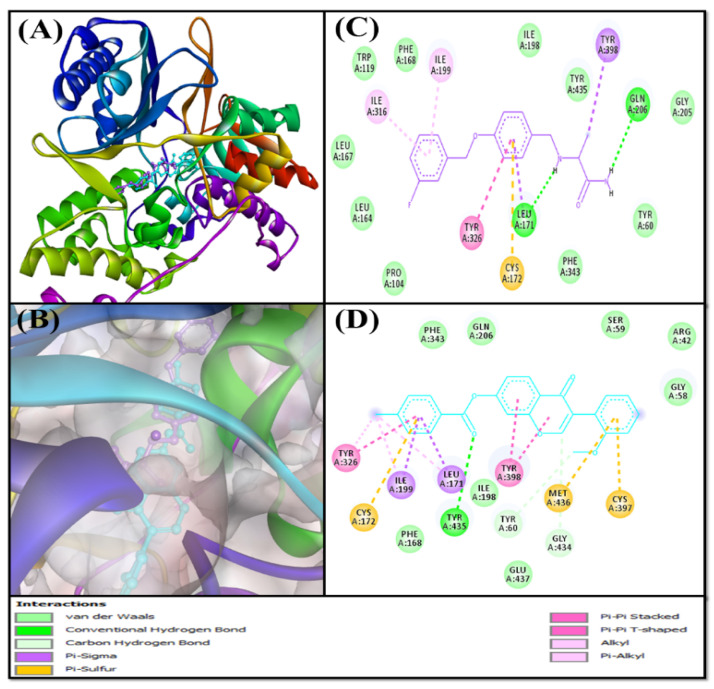
Interaction of target protein, MAO-B with F0850-4777 and their respective control ligands. (**A**) Position of F0850-4777 and Safinamide in MAO-B. (**B**) Interactions between MAO-B and Safinamide. (**C**) Superimposed image of F0850-4777 and Safinamide in MAO-B. (**D**) Interactions between MAO-B and F0850-4777.

Based on the virtual screening against a library of natural product-like compounds, F0850-4777 has been identified as the most potent inhibitor against multiple targets (AChE, BChE, MAO-A, and MAO-B) of AD. Further analysis by molecular docking between F0850-4777 and target proteins enabled us to closely examine the amino acid residues and the nature of interactions responsible for the formation of a stable protein–inhibitor complex. The interactions of F0850-4777 with the active site of AChE, BChE, MAO-A and MAO-B are shown in Figure 1, Figure 2, Figure 3 and Figure 4, respectively.

#### 2.3.1. Analysis of the Interaction between AChE and F0850-4777

Acetylcholinesterase (AChE) is an essential enzyme that catalyzes the hydrolysis of acetylcholine, which is critical for memory and cognition [34]. The inhibition of AChE activity is a major therapeutic intervention in the treatment of Alzheimer’s disease (AD), which is characterized by cholinergic deficiency. The majority of the drugs approved for the treatment of AD, such as Tacrine, donepezil, and rivastigmine, are AChE inhibitors [15,16,35]. The inhibitors of AChE activity bind to its catalytic active site (CAS), characterized by the presence of a long, narrow, and hydrophobic gorge, harboring a catalytic triad of Ser200, Glu327, and His440 [36]. The residues Trp84 and Phe330 play a significant role in stabilizing the transition state during the catalytic reaction. Furthermore, it has been recently demonstrated that a secondary noncholinergic function of AChE, associated with the peripheral anionic site (PAS), is involved in the pathogenesis of AD. PAS is formed by aromatic amino acid residues such as Tyr70, Asp72, Tyr121, Trp279, and Tyr334 lining the rim of the gorge [37]. Through its PAS, AChE co-localizes with Aβ peptide deposits in patients with AD and forms a stable Aβ-AChE complex, which in turn promotes fibrillogenesis and aggregation [38,39]. Thus, these observations suggest that both the CAS and PAS of AChE can be targeted as therapeutic interventions for AD.

In the present study, molecular docking analysis between AChE and F0850-4777 revealed that the ligand was bound to the central active site cavity of AChE (Figure 1). The binding pose of F0850-4777 at the active site of AChE was further compared with the binding mode of a control ligand, that is, Tacrine. Both F0850-4777 and Tacrine were found to occupy the same site located in the deep cavity of AChE (Figure 1A,B). The AChE-Tacrine complex was stabilized by one conventional hydrogen bond between the Lig:NH and Arg289:O atoms. In addition, five hydrophobic interactions (with Tyr121 and Trp279) and eight van der Waals’ interactions (Tyr70, Glu278, Leu282, Phe288, Phe290, Ser291, Phe331, and Tyr334) further stabilized the AChE-Tacrine complex (Figure 1C). Conversely, the AChE-F0580-4777 complex was mainly stabilized by hydrophobic interactions. F0850-4777 formed one Pi-Sigma interaction with Phe330, three Pi-Pi stacked interactions with Trp84 and Tyr121, five Pi-Pi T-shaped interactions with Tyr121, Phe330 and Tyr334, and two Pi-alkyl interactions with Tyr121 and Trp279 (Figure 1D and Table S1). In addition, several amino acid residues, such as Tyr70, Gly118, Glu199, Glu278, Phe290, Phe331, His440, Gly441, Ile439, and Tyr442 formed van der Waals’ interactions. It should be noted that F0850-4777 interacts with many CAS residues of AChE, including Trp84, Phe330, and His440, and PAS residues of AChE, such as Tyr121, Trp279, and Tyr334. Interestingly, the amino acid residues of AChE commonly interact with F0850-4777 as well as Tacrine includes Tyr121, Glu278, Trp279, Phe290, and Phe331. Moreover, the docking energy and the corresponding binding affinity were estimated to be −8.5 kcal mol^−1^ and 1.72 × 10^6^ M^−1^ for the AChE-Tacrine interaction, respectively, and −12.2 kcal mol^−1^ and 8.87 × 10^8^ M^−1^ for the AChE-F0850-4777 interaction, respectively. The binding affinity of F0850-4777 for AChE was approximately 515.7-fold higher than that of the control inhibitor Tacrine and RMSD value between best pose of Tacrine and F0850-4777 was found to be 1.345 Å.

#### 2.3.2. Analysis of the Interaction between BChE and F0850-4777

Butyrylcholinesterase (BChE), also known as pseudocholinesterase, is responsible for the hydrolysis of choline esters (e.g., butyrylcholine, succinylcholine, and acetylcholine) and non-choline esters (e.g., cocaine, acetylsalicylic acid, and heroin) [40,41]. BChE is a multifaceted enzyme expressed in different regions of neurons; it co-regulates cholinergic neurotransmission and is also partially involved in the development of the nervous system [42,43,44,45,46]. The fact that the biochemical properties of BChE are altered in AD makes it a potential target for use in therapeutic interventions [47,48,49,50]. Structurally and functionally, BChE is similar to AChE, which has a catalytic serine buried in a deep gorge. The catalytic triad of BChE is formed by Ser226, His438 and Glu352 [51]. The anionic site of BChE contains Trp82, which interacts with the cationic quaternary nitrogen of choline [52]. In addition, Asp70 and Tyr332 guide the positively charged substrates such as butyrylcholine to the active site located at the bottom of the gorge [53]. Furthermore, Leu286 and Val288, which line the acyl pocket within the active site gorge, hold the acyl group of choline in place during catalysis [52]. The acyl pocket of BChE is larger due to the presence of amino acid residues with smaller site chains (Leu286 and Val288) compared to the AChE acyl pocket lining Phe330.

Evaluating the interaction between BChE and F0850-4777 along with the control inhibitor (Tacrine) confirmed that both ligands occupied a similar position inside the binding cavity of BChE (Figure 2A,B). The BChE-Tacrine complex was stabilized by one conventional hydrogen bond between Lig:NH and the active site residue His438:O atoms. In addition, Tacrine formed four Pi-Pi stacked hydrophobic interactions with Trp82, and two Pi-Pi stacked interactions with His438. In addition, there were two Pi-alkyl (with Trp82 and Trp430) and one alkyl hydrophobic interaction with Ala328. Furthermore, the BChE and Tacrine complex was stabilized by six van der Waals interactions with Gly116, Glu197, Tyr332, Gly439, Tyr440, and Met437 (Figure 2C). Conversely, the BChE and F0850-4777 complex was stabilized by one carbon hydrogen bond with the Ser287:O atom, and seven hydrophobic interactions with Trp82, Pro285, and Tyr332 (Figure 2D and Table S2). In addition, several amino acid residues such as Asp70, Gly116, Tyr128, Glu197, Thr284, Ser287, Ala328, Phe329, His438, Gly439, and Tyr440 formed van der Waals’ interactions. It is worth noting that F0850-4777 interacts with some of the important amino acid residues of BChE such as Asp70, Trp82, and His438. Interestingly, the amino acid residues of BChE commonly engaged in interactions with F0850-4777 and Tacrine includes Trp82, Gly116, Glu197, Ala328, His438, Gly439, and Tyr440. Moreover, the docking energy and the corresponding binding affinity were estimated to be −8.4 kcal mol^−1^ and 1.45 × 10^6^ M^−1^ for the BChE-Tacrine interaction, respectively, and −10.7 kcal mol^−1^ and 7.04 × 10^7^ M^−1^ for the BChE-F0850-4777 interaction, respectively. We found that the binding affinity of F0850-4777 for BChE was approximately 48.6-fold higher than that of the control inhibitor Tacrine and RMSD value between best pose of Tacrine and F0850-4777 was found to be 1.401 Å.

#### 2.3.3. Analysis of the Interaction between Monoamine Oxidases and F0850-4777

Monoamine oxidases A and B (MAO-A and MAO-B) are located on the outer membrane of mitochondria. They catalyze the oxidation of amines to imines, which are then hydrolyzed non-enzymatically to the corresponding aldehydes or ketones [54]. MAO-A metabolizes serotonin, dopamine, and norepinephrine, whereas MAO-B oxidizes dopamine, benzylamine, and phenylethylamine [55,56]. MAO-B has also been reported to form a neurotoxin (1-methyl-4-phenyl-pyridinium), which causes Parkinson’s disease, from 1-methyl-4-phenyl-1,2,3,6-tetrahydropyridine [57]. Thus, monoamine oxidases are excellent targets for the development of novel therapeutics against Parkinson’s, Alzheimer’s, and other neurodegenerative diseases.

Structurally, MAO-A and MAO-B share 70% identical amino acid sequences, and both contain an FAD-binding domain, a substrate-binding domain, and a membrane-binding domain [58,59]. The catalytic sites of both monoamine oxidases are mainly hydrophobic and are lined with aromatic and aliphatic amino acid residues. A conserved lysine residue (Lys305 in MAO-A and Lys296 in MAO-B) interacts with a water molecule, which is attached to the N5-atom of the flavin co-factor [60]. The amino acid residues Tyr407 and Tyr444 in MAO-A, and Tyr398 and Tyr435 in MAO-B are conserved in all MAOs and are located on opposite sides of the covalently bound substrates and inhibitors [61,62]. It has been shown that these tyrosine residues orient the substrate for oxidation, or enhance the nucleophilicity of the amine [63].The selectivity of these enzymes in substrate binding sites is defined by the presence of Ile335 in MAO-A and Tyr326 in MAO-B [64]. Another difference between the two enzymes is the size of the substrate-binding site. In MAO-A, the volume of the substrate-binding site is 400 Å^3^, whereas in MAO-B, there is a smaller hydrophobic “entrance cavity” positioned between the surface and main substrate-binding site. Depending on the substrate, the two cavities in MAO-B are fused together because of the rotation in Ile199 to form a larger cavity of 400 Å^3^ [60].

##### Analysis of the Interaction between MAO-A and F0850-4777

Molecular docking analysis between MAO-A and F0850-4777 revealed that the ligand was bound to the central active site cavity of MAO-A (Figure 3). The binding pose of F0850-4777 at the active site of MAO-A was further compared with the binding mode of a control ligand, that is, Harmine. Both F0850-4777 and Harmine were found to occupy the same site located in the deep cavity of MAO-A (Figure 3A,B). The MAO-A-Harmine complex was stabilized by two carbon hydrogen bonds (Gly67:CA-Lig:O and Lig:C-Gly443:O), and five hydrophobic interactions with Tyr407 and Tyr444. In addition, Harmine formed eight van der Waals’ interactions with Ala68, Tyr69, Ile180, Asn181, Gln215, Met350, Phe352, and Met445 to further stabilize the MAO-A-Harmine complex (Figure 3C). Conversely, the MAO-A and F0850-4777 complex was stabilized by one conventional hydrogen bond (Tyr407:HH-Lig:O) and one carbon hydrogen bond (Lig:C-Tyr69:O). In addition, F0850-4777 formed three Pi-Pi stacks (Tyr407 and Tyr444), and seven Pi-alkyl hydrophobic interactions (with Val210, Cys323, Ile335, Leu337, and Met445). In addition, F0850-4777 formed two Pi-Sulfur interactions with Cys323 and Cys406 (Figure 3D and Table S3). Furthermore, the MAO-A-F0850-4777 complex was stabilized by van der Waals’ interactions with several amino acid residues such as Arg51, Thr52, Gly67, Ala68, Ile180, Phe208, Gln215, Met350, Phe352, Gly443, and Glu446. Interestingly, the amino acid residues of MAO-A commonly interacted with F0850-4777 and Harmine with Gly67, Ala68, Tyr69, Ile180, Gln215, Met350, Phe352, Tyr407, Gly443, and Tyr444. Moreover, the docking energy and the corresponding binding affinity were estimated to be −8.7 kcal mol^−1^ and 2.40 × 10^6^ M^−1^ for the MAO-A-Harmine interaction, respectively, and −13.6 kcal mol^−1^ and 9.44 × 10^9^ M^−1^ for the MAO-A-F0850-4777 interaction, respectively. The binding affinity of F0850-4777 for MAO-A was approximately 3933.33-fold higher than that of the control inhibitor Harmine and RMSD value between best pose of Harmine and F0850-4777 was found to be 1.840 Å.

##### Analysis of the Interaction between MAO-B and F0850-4777

An insight into the interaction between MAO-B and F0850-4777 along with the control inhibitor (Sulfinamide) confirmed that both the ligands occupied a similar pose inside the binding cavity of MAO-B (Figure 4A,B). The MAO-B-Sulfinamide complex was stabilized by two conventional hydrogen bonds (Lig:H-Leu171:O, and Lig:H-Gln206:OE1). In addition, Sulfinamide formed two Pi-Sigma hydrophobic interactions with Leu171:CD2, and Tyr398 along with one Pi-Pi T-shaped interaction with Tyr326, and two Pi-alkyl interactions with Ile199 and Ile316. In addition, there was one Pi-Sulfur interaction with Cys172:SG. Furthermore, the MAO-B and Sulfinamide complex was stabilized by ten van der Waals’ interactions with Tyr60, Pro104, Trp119, Leu164, Leu167, Phe168, Ile198, Gly205, Phe343, and Tyr435 (Figure 4C). Conversely, the MAO-B and F0850-4777 complex was stabilized by one conventional hydrogen bond with Tyr435, and two carbon hydrogen bonds with Tyr60, and Gly434 (Figure 4D and Table S4). In addition, F0850-4777 formed two Pi-Sigma hydrophobic interactions (with Leu171:CD2 and Ile199:CA), two Pi-Pi-stacked interactions with Tyr398, one Pi-Pi T-shaped interaction with Tyr326, two Pi-Pi stacked interactions with Tyr398, one Pi-alkyl interaction with Tyr326, and two alkyl interactions with Leu171 and Ile199. Moreover, F0850-4777 also formed three Pi-Sulfur interactions with Cys172:SG, Cys397:SG, and Met436:SD residues. Several amino acid residues, such as Arg42, Gly58, Ser59, Phe168, Ile198, Gln206, Phe343, and Glu437, were found to form van der Waals’ interactions. Interestingly, the amino acid residues of MAO-B commonly engaged in the interaction with F0850-4777 as well as Sulfinamide were Tyr60, Phe168, Leu171, Cys172, Ile198, Ile199, Gln206, Tyr326, Phe343, Tyr398, and Tyr435. Moreover, the docking energy and the corresponding binding affinity were estimated to be −9.5 kcal mol^−1^ and 9.28 × 10^6^ M^−1^ for the MAO-B-Sulfinamide interaction, respectively, and −12.5 kcal mol^−1^ and 1.47 × 10^9^ M^−1^ for the MAO-B-F0850-4777 interaction, respectively. We found that the binding affinity of F0850-4777 for MAO-B was approximately 158.41-fold higher than the control inhibitor Sulfinamide and RMSD value between best pose of Sulfinamide and F0850-4777 was found to be 2.880 Å.

### 2.4. Analysis of Molecular Dynamics Simulation

#### 2.4.1. Root Mean Square Deviation (RMSD) Analysis

**Figure 5:**
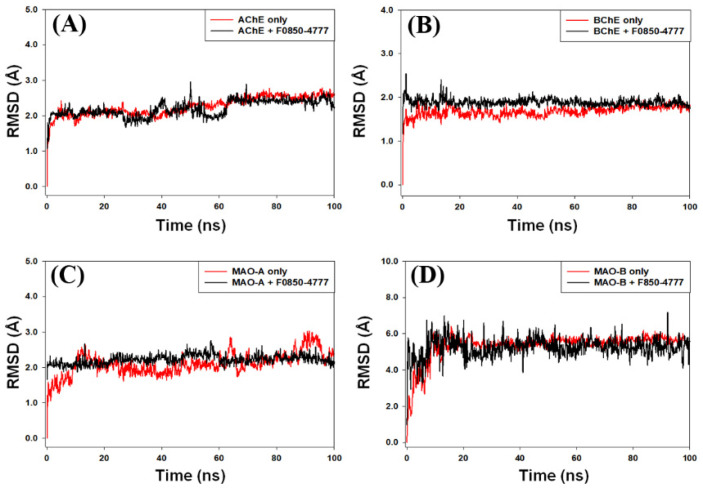
Behavior of root mean square deviation (RMSD) of (**A**) AChE, (**B**) BChE, (**C**) MAO-A, and (**D**) MAO-B alone or in complex with F0850-4777.

In molecular dynamics simulations, the measurement of RMSD provides an estimate of the stability and dynamic nature of the protein–ligand complex. RMSD is measured as the deviation in the structure of a protein or protein–ligand complex from its initial pose, which eventually gives an insight into the stability of protein–ligand complex during simulation. Here, we report the behavior of RMSD of AChE, BChE, MAO-A, and MAO-B alone or in complex with F0850-4777 during molecular dynamics simulation under physiological conditions (Figure 5). The RMSD of AChE and BChE in the absence of F0850-4777 increased sharply for the initial 2 ns, and then stayed consistent for the rest of simulation, while the RMSDs of AChE-F0850-4777 and BChE-F0850-4777 complexes fluctuated within the acceptable limits throughout the simulation (Figure 5A,B). Moreover, the RMSD of MAO-A and MAO-B in the absence of F0850-4777 fluctuated slightly during 0–15 ns, and thereafter remained constant for the remaining simulation time, while the RMSDs of MAO-A and MAO-B in the presence of F0850-4777 followed a consistent path throughout the simulation (Figure 5C,D). The average RMSD values of AChE, BChE, MAO-A, and MAO-B in the absence and presence of F0850-4777 estimated during 20–100 ns were 2.33 ± 0.16 Å, 2.08 ± 0.12 Å, 1.70 ± 0.09 Å, 1.98 ± 0.10 Å, 2.15 ± 0.11 Å, 2.06 ± 0.07 Å, 5.81 ± 0.34 Å, and 5.33 ± 0.41 Å, respectively. It is worth noting that none of the fluctuations in RMSD were more than the acceptable limit of 2.0 Å. These results suggest that the overall structures of target enzymes (AChE, BChE, MAO-A, and MAO-B) did not change significantly due to the binding of F0850-4777, and the protein–ligand complexes remained stable throughout the simulation.

#### 2.4.2. Root Mean Square Fluctuation (RMSF) Analysis

**Figure 6:**
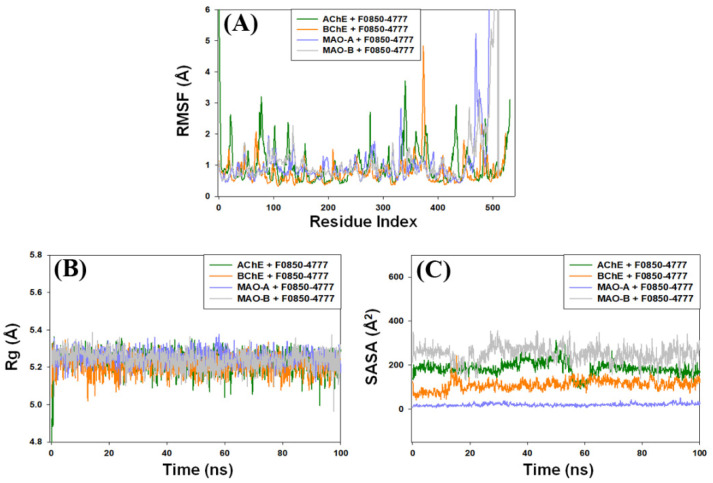
(**A**) Average root mean square fluctuation (RMSF) values of AChE, BChE, MAO-A, and MAO-B in the presence of F0850-4777; (**B**) the variation in Rg of F0850-4777 bound with different proteins (AChE, BChE, MAO-A, and MAO-B); (**C**) SASA of target proteins AChE, BChE, MAO-A, and MAO-B bound to F0850-4777.

During molecular dynamics simulation, the measurement of protein RMSF is significant to access the local conformational changes in the side chains of a protein occurred due to ligand binding. In this study, we monitored the RMSF of F0850-4777 bound with AChE, BChE, MAO-A, and MAO-B (Figure 6A). It is generally observed that the residues at the N and C-terminal or loop regions display higher fluctuations. The average RMSF values of AChE, BChE, MAO-A, and MAO-B in the presence of F0850-4777 were 0.98 ± 0.06 Å, 0.79 ± 0.04 Å, 1.04 ± 0.09 Å, and 1.16 ± 0.11 Å, respectively. These results indicate that the RMSF of AChE, BChE, MAO-A, and MAO-B did not deviate significantly in the presence of F0850-4777 and the average values remained within the acceptable limits, thereby indicating that the overall conformation of target proteins was conserved.

#### 2.4.3. Analysis of Radius of Gyration (Rg) and Solvent Accessible Surface Area (SASA)

The dependency of radius of gyration (Rg) and solvent accessible surface area (SASA) of a ligand on simulation time give information about the behavior of the ligand inside the binding pocket of the enzyme. The Rg values describe the RMSD of an atom’s width from the common center of mass. The Rg may also be used to determine whether the complex remains folded during the MD simulation. The variation in Rg of F0850-4777 bound with different proteins (AChE, BChE, MAO-A, and MAO-B) as a function of simulation time is presented in Figure 6B. The results show that the Rg values of different protein–ligand systems fluctuated within the acceptable limit throughout the simulation. The average Rg values of AChE, BChE, MAO-A, and MAO-B bound with F0850-4777 were estimated as 5.23 ± 0.28 Å, 5.21 ± 0.24 Å, 5.25 ± 0.19 Å, and 5.24 ± 0.27 Å, respectively.

The solvent accessible surface area (SASA) measures the exposure of a protein to the solvent, thereby indicating if the protein is in native conformation upon the binding of a ligand. Here, we measured SASA of target proteins AChE, BChE, MAO-A, and MAO-B bound to F0850-4777 (Figure 6C). It is evident that the SASA of AChE-F0850-4777, BChE-F0850-4777, MAO-A-F0850-4777, and MAO-B-F0850-4777 complexes varied slightly with the acceptable limits. The average SASA values of F0850-4777 bound with AChE, BChE, MAO-A, and MAO-B were 185.4 ± 5.63 Å^2^, 110.0 ± 4.39 Å^2^, 19.6 ± 1.01 Å^2^, and 250.7 ± 4.73 Å^2^, respectively. These results suggest that F0850-4777 remained inside the binding cavity of AChE, BChE, MAO-A, and MAO-B in a stable conformation.

#### 2.4.4. Secondary Structure Analysis

**Figure 7:**
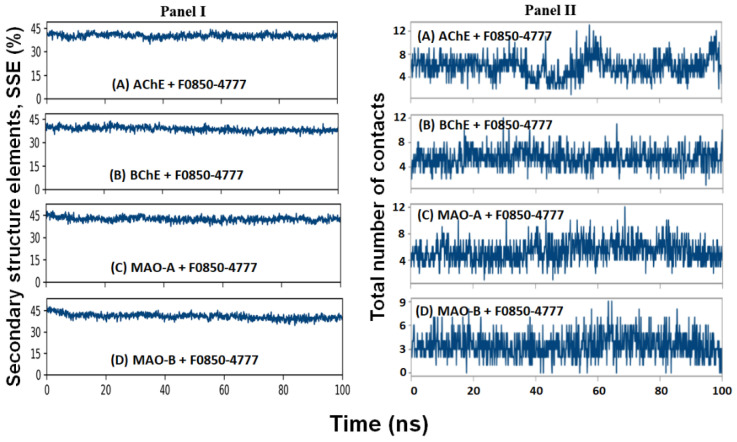
(Panel I) Variation in total secondary structural elements (α-helix + β-sheet) of AChE, BChE, MAO-A and MAO-B in the presence of F0850-4777 during simulation. (Panel II) Total number of contacts formed between F0850-4777 and (**A**) AChE, (**B**) BChE, (**C**) MAO-A, and (**D**) MAO-B during simulation.

The interaction between a ligand and protein often leads to changes in the protein’s secondary structural elements (SSE). Thus, evaluating the variation in SSE during simulation is critical to verify the establishment of a stable complex between the ligand and protein. In this study, we monitored the variation in the total SSE (α-helix + β-sheet) of AChE, BChE, MAO-A, and MAO-B in the presence of F0850-4777 during the simulation (Figure 7: Panel I). We found that the total SSE of AChE, BChE, MAO-A, and MAO-B in complex with F0850-4777 was 40.09 ± 2.62 % (α-helix: 26.92 ± 2.41 % and β-sheets: 13.17 ± 1.03 %), 38.71 ± 3.43 % (α-helix: 26.57 ± 2.76 % and β-sheets: 12.14 ± 2.04 %), 42.33 ± 3.12 % (α-helix: 25.81 ± 2.59 % and β-sheets: 16.52 ± 1.74 %), and 40.87 ± 2.63 % (α-helix: 25.94 ± 2.12 % and β-sheets: 14.93 ± 1.55 %), respectively. It is worth noting that the SSE of all the targeted proteins in combination with F0850-4777 remained consistent throughout the simulation, suggesting a stable interaction between proteins and ligand.

#### 2.4.5. Contact between F0850-4777 and Target Proteins

The formation of a stable protein and ligand complex was established by determining the total number of contacts formed between them during the simulation (Figure 7: Panel II). It is clear that during simulation, the total number of contacts between F0850-4777 and AChE, BChE, MAO-A, and MAO-B varied between 2–13, 2–13, 1–12, and 0–9, respectively. On average, AChE, BChE, MAO-A, and MAO-B formed 7, 6, 6, and 4 contacts with F0850-4777 respectively. These results confirmed that F0850-4777 remained in the binding pockets of target proteins throughout the simulation.

**Figure 8:**
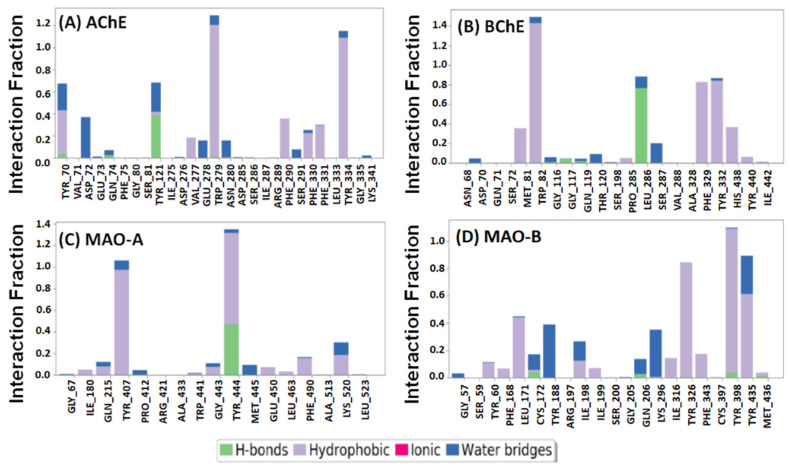
Interactions of F0850-4777 with (**A**) AChE, (**B**) BChE, (**C**) MAO-A, and (**D**) MAO-B.

The overall interaction between target proteins and F0850-4777 over the simulation was also determined and represented in Figure 8. We found that the interaction between AChE and F0850-4777 through amino acid residues such as Tyr70, Asp72, Tyr121, Trp279, Phe290, Phe330, Phe331, and Tyr334 remained consistent throughout the MD simulation (Figure 8A). Similarly, the interaction between BChE and F0850-4777 through Met81, Trp82, Leu286, Phe329, Tyr332, and His438 remain intact during the MD simulation (Figure 8B). The amino acid residues of MAO-A forming a stable contact with F850-4777 during MD simulation were Tyr407, Tyr444, and Lys520 (Figure 8C). Furthermore, the interaction between MAO-B and F850-4777 through amino acid residues such as Leu171, Tyr188, Ile198, Gln206, Lys296, Tyr326, Tyr398, and Tyr435 remain stable throughout the MD simulation (Figure 8D). Furthermore, the stability of ligand inside the binding pocket of their respective protein targets was evaluated by monitoring RMSF of the ligand, as shown in Figure S2. It was observed that none of the RMSF values exceeded 2 Å, confirming the stability of the protein–ligand complexes.

#### 2.4.6. Analysis of Free Energy (Prime-MM/GBSA) Calculations

Free energy calculation by Prime-MM/GBSA is an accurate method to evaluate protein–ligand stability in the presence of a solvent. In this study, the Prime-MM/GBSA of targeted proteins and F0850-4777 was calculated and the results are presented in Table 4. As evident from Table 4, AChE has the lowest ΔG_Bind_ energy (−30.35 ± 3.28 kcal mol^−1^), followed by MAO-B (−29.38 ± 2.99 kcal mol^−1^), BChE (−23.39 ± 3.07 kcal mol^−1^), and MAO-A (−20.64 ± 2.93 kcal mol^−1^). Principally, van der Waals’ energy (ΔG_vdW_) and non-polar solvation or lipophilic energy (ΔG_Sol_Lipo_) contribute favorably towards the formation of a stable protein–ligand complex, while covalent (ΔG_Covalent_) and polar solvation energies (ΔG_Solv_ or ΔG_SolGB_) oppose the formation of a stable protein–ligand complex.

## 3. Materials and Methods

### 3.1. Hardware and Software Used

The three-dimensional coordinates of the target enzymes (AChE, BChE, MAO-A and MAO-B) were downloaded from the PDB database (http://www.rcsb.org/pdb/). PyRx-Python Prescription 0.8 [65] using Autodock-Vina [66] with the Lamarckian genetic algorithm as a scoring function was used for molecular docking. Molecular interactions for the best scoring ligand were separately analyzed by Discovery Studio 2020 (BIOVIA) software package. Molecular dynamics was performed on an Intel Xenon workstation- E3-1245-8C, 3.50 GHz processor with 28 GB RAM. The workstation was powered by a NVIDIA Quadro P5000 GPU card. Desmond (Shchrodinger-2020, LLC, NY, USA) was employed to conduct molecular dynamics simulation.

### 3.2. Ligands Preparation

The natural product-like compound library from Life Chemicals (www.lifechemicals.com) was screened to identify novel inhibitors of the targeted enzymes. The library contains 2029 compounds (accessed November 2020). The ligands were downloaded in sdf format and converted to Autodock suitable pdbqt format along with density function theory (DFT) optimization of the minimum energy conformer using the inbuilt function in PyRx. The energy of all the ligands was minimized in PyRx using universal force field (UFF).

### 3.3. Protein Target Preparation

The three-dimensional coordinates of AChE (PDB Id: 1ACJ), BChE (PDB Id: 4BDS), MAO-A (PDB Id: 2Z5X), and MAO-B (PDB Id: 2V5Z) were downloaded from the PDB database (http://www.rcsb.org/pdb/). The target proteins were prepared for molecular docking by native ligand and non-essential water molecules, assigning hydrogen polarities, calculating Gasteiger charges to protein structures, and converting protein structures from the pdb file format to pdbqt format. Energy minimization and geometry optimization of all structures were performed using a built-in tool in PyRx. Subsequently, the targeted proteins were exploited for the binding pockets from crystal structures and were further evaluated using the Uniprot.

### 3.4. Molecular Docking

Molecular docking was performed using the PyRx-Python 0.8 virtual screening tool coupled with AutoDock 4.2, employing the Lamarckian genetic algorithm method [67,68]. All of the ligands were individually docked with each of the targeted enzymes as separate docking runs. The grid dimensions for AChE were selected through discovery studio visualizer (BIOVIA) from the attributes of docked ligand (control inhibitor) in its specific target protein and set to 60 × 60 × 60 Å centered at 4.6 × 70.1 × 65.9 Å, whereas grid dimensions for BChE, MAO-A, and MAO-B were set to 33 × 33 × 33 Å centered at 140.1 × 122.2 × 38.9 Å, 126 × 126 × 126 Å centered at 30.9 × 28.8 × 14.9 Å, and 126 × 126 × 126 Å centered at 53.5 × 147.8 × 24.4 Å, respectively, as discussed in previous reports [69,70]. The results were clustered according to the root-mean-square deviation (RMSD) criterion and in the current study we selected the ligands with lower than 3Å RMSD modes between the best docked pose of natural product-like compound and reference inhibitor. The docking was performed with the “exhaustiveness” set to 8. All other docking parameters were set to the default values of the software. The binding affinity (K_d_) of ligands for the target enzyme was calculated from the binding energy (ΔG) using the following relation [71,72]:

(1) $$\Delta G=-{\mathrm{RT}\;\mathrm{lnK}}_{d}$$

where R and T were the Boltzmann’s gas constant and temperature respectfully.

The ligands with the minimum binding energy were selected for further analysis. The best pose of each “protein–ligand complex” was generated and analyzed using Discovery Studio 2020 (BIOVIA).

### 3.5. Prediction of Physicochemical, Pharmacokinetics Properties, Drug-Likeness, and Toxicity Potentials

About the 10 top best hits from the total 2029 compounds were analyzed against cholinesterases and monoamine oxidases were assessed for their physicochemical properties, drug-likeness, and pharmacokinetics using the SwissADME (http://www.swissadme.ch) web-based tool. The tool was used to assess the molecular weight, the number of hydrogen bond donors and acceptors, rotatable bonds, cLogP value, topological polar surface area, Lipinski’s rule violation, human gastrointestinal absorption (HIA), and blood–brain barrier (BBB) permeation to finalize the bioactive compound for further computational analysis [33]. The fraction of sp^3^ carbon atoms (Fsp^3^), a key factor for drug-likeness, was also analyzed through SwissADME [73]. Moreover, various aspects and effects of the toxicity, including the tumorigenicity, mutagenicity, and irritability of the selected compounds, were also tested using the Orisis Datawarrior tool [74]. In the Orisis Datawarrior tool’s analysis, the predicted toxicity values were depended on comparing the precalculated investigated molecules with the tested molecule’s structures.

### 3.6. Molecular Dynamics (MD) Simulation

MD simulation of the best scoring ligand was performed in complex with their respective targeted enzymes (AChE, BChE, MAO-A, and MAO-B) in triplicates using “Desmond (Schrodinger-2020, LLC, NY, USA)” as described earlier [26,75]. The protein–ligand complex obtained in the AutoDock Vina is imported to the Maestro interface of the Schrodinger’s software. Prior to MD simulation, the protein–ligand complex was optimized by adding missing hydrogen atoms, assigning proper protonation state of the ligand and other parameters using Protein preparation wizard. The protein–ligand complex was placed at the center of an orthorhombic box, keeping a distance of at least 10 Å from the sides of the box. TIP3P water molecules were added to solvate the simulation box, and proper counterions were also added to neutralize the system. The physiological conditions were mimicked by adding 150 mM NaCl. The energy of the whole system was minimized with 2000 iteration and convergence criteria of 1 kcal/mol/Å, using OPLS3e forcefield. The production MD simulation run was performed for 100 ns employing NPT ensemble at 298 K and 1 bar. Temperature and pressure were maintained with the help of Nose-Hoover Chain thermostat and Matrtyna–Tobias–Klein barostate [76,77]. A 2 fs time step was fixed, and at every 10 ps, energies and structures were documented in the trajectory. The parameters such as root mean square deviation (RMSD), root mean square fluctuation (RMSF), radius of gyration (Rg), solvent accessible surface area (SASA), secondary structure analysis, and protein–ligand interactions were analyzed to establish the stability of protein–ligand complexes. The results are presented as mean ± standard deviation of the three independent experiments.

### 3.7. Free Energy (Prime-MM/GBSA) Calculations

The binding free energy of each protein–ligand complex was estimated using Prime module (Schrodinger, LLC, NY, USA) employing the MM-GBSA approach, as described previously [75,78]. In this approach, free energy was computed on the final 10 ns MD simulation trajectories, once equilibration had been reached. Briefly, first, the docked complexes were subjected to local optimization through molecular mechanics (MM) in Prime, and then their energies were minimized with OPLS-AA (2005) force field with the generalized Born surface area (GBSA) continuum solvent model. The binding free energy (ΔG_Bind_) is estimated as:

(2) $$\Delta G_{\mathrm{Bind}}=\Delta E_{\mathrm{MM}}+\Delta G_{\mathrm{Solv}\_\mathrm{GB}}+\Delta G_{\mathrm{SA}}$$

(3) $$\Delta E_{\mathrm{MM}}=E_{\mathrm{Complex}}-\left(E_{\mathrm{Protein}}+E_{\mathrm{Ligand}}\right)$$

where E_Complex_, E_Protein_, and E_Ligand_ are the respective values of minimized energies of protein–ligand complex, protein, and ligand.

(4) $$\Delta G_{\mathrm{Solv}\_\mathrm{GB}}=G_{\mathrm{Solv}\_\mathrm{GB}\;\left(\mathrm{Complex}\right)}-\left(G_{\mathrm{Solv}\_\mathrm{GB}\;\left(\mathrm{Protein}\right)}+G_{\mathrm{Solv}\_\mathrm{GB}\;\left(\mathrm{Ligand}\right)}\right)$$

where G_Solv_GB (Complex)_, G_Solv_GB (Protein)_, and G_Solv_GB (Ligand)_ are the respective values of free energies of solvation of protein–ligand complex, protein, and ligand.

(5) $$\Delta G_{\mathrm{SA}}=G_{\mathrm{SA}\;\left(\mathrm{Complex}\right)}-\left(G_{\mathrm{SA}\;\left(\mathrm{Protein}\right)}-G_{\mathrm{SA}\left(\mathrm{Ligand}\right)}\right)$$

where G_SA (Complex)_, G_SA (Protein)_, and G_SA (Ligand)_ are the respective values of surface area energies of protein–ligand complex, protein, and ligand.

In the Prime-MM/GBSA method, the free energy is calculated as:

(6) $$\Delta G_{\mathrm{Bind}}=\Delta G_{\mathrm{Coulomb}}+\Delta G_{\mathrm{vdW}}+\Delta G_{\mathrm{Covalent}}+\Delta G_{H-\mathrm{bond}}+\Delta G_{\mathrm{Sol}\_\mathrm{Lipo}}\;+\Delta G_{\mathrm{Solv}\_\mathrm{GB}}+\Delta G_{\mathrm{Packing}}+\Delta G_{\mathrm{Self}-\mathrm{contact}}$$

## 4. Conclusions

Using high-throughput screening and the molecular dynamics simulation study, we concluded that the F0850-4777 compound, out of 2029 natural product-like compounds, showed the best binding affinity against all the four targets and exhibited the finest drug-likeness, pharmacokinetics and physiological properties which can cross the BBB as well as high absorption through GI tract with non-toxic potential. The findings of this study suggest that the F0850-4777 can be a potential candidate against multiple-targets of two pathophysiological pathways pertaining to AD. In this study, neuroprotective potentials of candidate drug were explored only via in silico approaches and open the window for confirmation of its therapeutic efficacy through in vitro and in vivo systems.

## Figures and Tables

**Table 1:** Molecular docking scores of best hit natural product-like compounds against AChE, BChE, MAO-A and MAO-B.

S. No.	ID Number	Targets/ Formula	Docking Energy (kcal/mol)			
			AChE (1acj)	BChE (4bds)	MAO-A (2z5x)	MAO-B (2v5z)
1	F0870-0001	C_24_H_15_NO_6_	−12.9	−12.6	−11.5	−13.6
2	F1094-0205	C_26_H_23_NO_4_	−12.9	−11	−10.8	−12.6
3	F3293-0320	C_22_H_13_NO_7_	−12.4	−11.1	−12.3	−13.4
4	F1094-0201	C_26_H_19_NO_4_	−12.3	−11.2	−12.3	−11.5
5	F0850-4777	C_24_H_18_O_5_	−12.2	−10.7	−13.6	−12.5
6	F3385-6048	C_27_H_21_NO_8_	−12.2	−11.1	−13.2	−12.6
7	F1094-0200	C_25_H_17_NO_4_	−12.1	−11.2	−11	−13.2
8	F1865-0198	C_23_H_15_NO_6_	−12	−10.9	−12.6	−13.3
9	F3139-1101	C_24_H_16_O_4_	−12	−10.3	−12.4	−13.6
10	F3139-1218	C_26_H_18_O_6_	−11.8	−10.4	−12.9	−13.3
11	Tacrine	C_13_H_14_N_2_	−8.5	−8.4	ND	ND
12	Harmine	C_13_H_12_N_2_O	ND	ND	−8.7	ND
13	Safinamide	C_17_H_19_FN_2_O_2_	ND	ND	ND	−9.5

ND: Not determined.

**Table 2:** Physicochemical properties, drug-likeness, and pharmacokinetics of best hit natural product-like compounds.

		Pharmacokinetics		Physicochemical Properties						Drug-Likeness	
S. No.	ID Number	BBB-P	GI-A	MW	Clog-P	HBA	HBD	RB	TPSA	L-V	FSP^3^
1	F0870-0001	NO	High	413.37	3.29	7	2	3	113.77	0	0.04
2	F1094-0205	YES	High	413.47	6.32	5	0	3	59.75	0	0.3
3	F3293-0320	NO	High	403.34	3.25	7	0	6	119.4	0	0.04
4	F1094-0201	YES	High	409.43	5.79	5	0	2	59.75	0	0.15
5	F0850-4777	YES	High	386.39	4.51	5	0	5	65.74	0	0.08
6	F3385-6048	NO	High	487.46	4.99	9	0	6	96.67	0	0.18
7	F1094-0200	YES	High	395.4	5.48	5	0	2	59.75	0	0.12
8	F1865-0198	NO	High	401.37	5.34	6	0	5	102.37	0	0.04
9	F3139-1101	YES	High	368.38	5.12	4	0	5	56.51	0	0
10	F3139-1218	NO	High	426.42	4.45	6	0	5	78.88	0	0.07

Molecular weight (MW), number of hydrogen bond donors (HBD), number of hydrogen bond acceptors (HBA), rotatable bonds (RB), cLogP value (clogP), topological polar surface area (TPSA), Lipinski’s rule violation (L-V), human gastrointestinal absorption (GI-A), blood–brain barrier permeation (BBB-P) and fraction Csp3 (FSP^3^).

**Table 3:** Toxicity potential of 10 hits compounds.

S. No.	Compound	Mutagenic	Tumorigenic	Reproductive Effect	Irritant
1	F0850-4777	None	None	None	None
2	F0870-0001	None	None	Low	None
3	F1094-0200	None	None	High	None
4	F1094-0201	None	None	High	None
5	F1094-0205	None	None	High	None
6	F1865-0198	None	None	High	None
7	F3139-1101	None	None	None	High
8	F3139-1218	None	None	High	None
9	F3293-0320	None	None	None	None
10	F3385-6048	None	None	None	None

Toxicity assessment has been performed by DataWarrior tool.

**Table 4:** Free energy calculation of targeted proteins and F0850-4777 complexes using Prime/MM-GBSA.

Proteins	ΔE_MM_			ΔG_Solv_ or ΔG_SolGB_	ΔG_Self-contact_	ΔG_H-bond_	ΔG_SA_ or ΔG_Sol_Lipo_	ΔG_Packing_	ΔG or ΔG_Bind_
	ΔG_Coulomb_	ΔG_vdW_	ΔG_Covalent_						
AChE	1.25 ± 0.87	−19.24 ± 1.52	0.65 ± 0.05	6.18 ± 0.54	0	−0.16 ± 0.04	−15.81 ± 1.22	−3.22 ± 0.28	−30.35 ± 3.28
BChE	−0.54 ± 0.04	−20.17 ± 1.41	1.16 ± 0.06	9.65 ± 0.69	0	0	−13.49 ± 1.07	0	−23.39 ± 3.07
MAO-A	−6.14 ± 0.39	−17.21 ± 1.19	3.79 ± 0.06	12.71 ± 1.06	0	−1.20 ± 0.03	−11.65 ± 0.08	−0.94 ± 0.03	−20.64 ± 2.93
MAO-B	−3.97 ± 0.23	−17.80 ± 1.14	−0.05 ± 0.01	9.39 ± 0.57	0	−0.18 v	−16.28 ± 1.09	−0.49 ± 0.02	−29.38 ± 2.99

All the energies are in kcal mol^−1^. ΔE_MM_, ΔG_Coulomb_, ΔG_vdW_, ΔG_Covalent_, ΔG_Solv_ or ΔG_SolGB_, ΔG_Self-contact_, ΔG_H-bond_, ΔG_SA_ or ΔG_Sol_Lipo_, and ΔG or ΔG_Bind_ stands for minimized molecular mechanics energy, coulomb energy, van der Waals’ energy, covalent binding energy, solvation energy, energy due to self contact, energy due to H-bonds, lipophilic energy, and binding energy, respectively.

## Data Availability

Not applicable.

## Supplementary Materials

The following are available online at https://www.mdpi.com/article/10.3390/ph14090937/s1, Table S1: Molecular docking parameters for the interaction of target protein, acetylcholinesterase with F0850-4777 and their respective control ligands; Table S2: Molecular docking parameters for the interaction of target protein, butyrylcholinesterase with F0850-4777 and their respective control ligands; Table S3: Molecular docking parameters for the interaction of target protein, monoamine oxidase-A with F0850-4777 and their respective control ligands; Table S4: Molecular docking parameters for the interaction of target protein, monoamine oxidase-B with F0850-4777 and their respective control ligands; Figure S1: (A) structure of F0850-4777 (3-(2-methoxyphenyl)-4-oxo-4H-chromen-7-yl 4-methylbenzoate), (B) acceptable range (pink color region) for pharmacokinetics properties of F0850-4777, (C) description of BOILED-Egg image for F0850-4777 to predict gastrointestinal absorption (HIA) and brain penetration (BBB); Figure S2. RMSF of ligand (F0850-4777) inside the binding pocket of their respective protein targets.
