# Supplementary material for: High-Throughput Screening and Molecular Dynamics Simulation of Natural Product-like Compounds against Alzheimer’s Disease through Multitarget Approach
Source: Pharmaceuticals (Basel). 2021 Sep 18;14(9):937. doi: 10.3390/ph14090937 (PMC8466787; doi:10.3390/ph14090937)
Supplement: Supplementary file 1 [file pharmaceuticals-14-00937-s001.zip › pharmaceuticals-1371918-supplementary.pdf]

Supplementary files

High-throughput screening and molecular dynamics simulation of natural product-like compounds against Alzheimer's disease through multitarget approach

Danish Iqbal [1, *], Md Tabish Rehman [2], Abdulaziz Bin Dukhyil [1], Syed Mohd Danish Rizvi [3], Mohamed F AlAjmi [2], Bader Mohammed Alshehri [1], Saeed Banawas [1,4,5], M. Salman Khan [6], Wael Alturaiki [1], and Mohammed Alsaweed [1]

[1] Department of Medical Laboratory Sciences, College of Applied Medical Sciences, Majmaah University, Majmaah, 11952, Saudi Arabia; da.mohammed@mu.edu.sa (D.I.), a.dukhyil@mu.edu.sa (A.B.D.), w.alturaiki@mu.edu.sa (W.T.), m.alsaweed@mu.edu.sa (M.A), b.alshehri@mu.edu.sa (B.M.A.)
[2] Department of Pharmacognosy, College of Pharmacy, King Saud University, Riyadh 11451, Saudi Arabia; mrehman@ksu.edu.sa (M.T.R), malajmii@ksu.edu.sa (M.F.A)
[3] Department of Pharmaceutics, College of Pharmacy, University of Hail, Hail P.O. Box 2240, Saudi Arabia; sm.danish@uoh.edu.sa (S.M.D.R.)
[4] Health and Basic Sciences Research Center, Majmaah University, Al Majmaah 15341, Saudi Arabia; s.banawas@mu.edu.sa (S.B.)
[5] Department of Biomedical Sciences, Oregon State University, Corvallis, Oregon 97331, United States
[6] Clinical Biochemistry & Natural Product research laboratory, Department of Biosciences, Integral University, lucknow-226026, U.P., India; contactskhan@gmail.com (M.S.K.)

* Correspondence: da.mohammed@mu.edu.sa

Table of Content:

Figure S1: (A) Structure of F0850-4777 (3-(2-methoxyphenyl)-4-oxo-4H-chromen-7-yl 4-methylbenzoate), (B) Acceptable range (pink color region) for pharmacokinetics properties of F0850-4777, (C) Description about BOILED-Egg image for F0850-4777 to predict gastrointestinal absorption (HIA) and brain penetration (BBB).
Figure S2. RMSF of ligand (F0850-4777) inside the binding pocket of their respective protein targets
Table S1: Molecular docking parameters for the interaction of target protein, acetylcholinesterase with F0850-4777 and their respective control ligands.
Table S2: Molecular docking parameters for the interaction of target protein, butyrylcholinesterase with F0850-4777 and their respective control ligands.
Table S3: Molecular docking parameters for the interaction of target protein, monoamine oxidase-A with F0850-4777 and their respective control ligands.
Table S4: Molecular docking parameters for the interaction of target protein, monoamine oxidase-B with F0850-4777 and their respective control ligands.

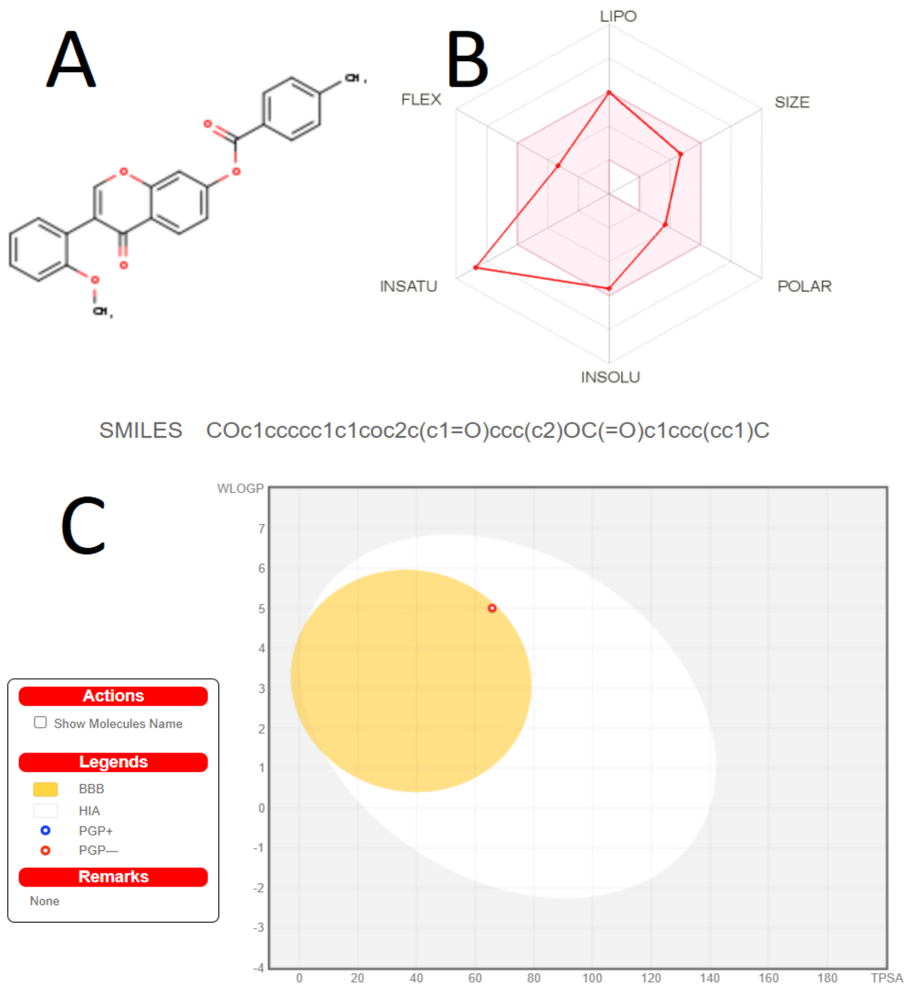

Figure S1: (A) Structure of F0850-4777 (3-(2-methoxyphenyl)-4-oxo-4H-chromen-7-yl 4-methylbenzoate), (B) Acceptable range (pink color region) for pharmacokinetics properties of F0850-4777, (C) Description about BOILED-Egg image for F0850-4777 to predict gastrointestinal absorption (HIA) and brain penetration (BBB).

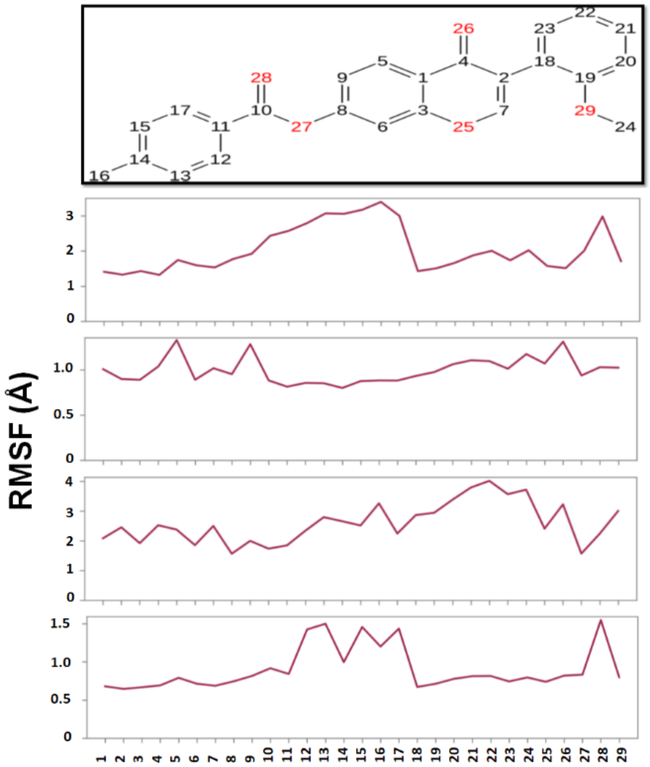

Figure S2. RMSF of ligand (F0850-4777) inside the binding pocket of their respective protein targets

Table S1: Molecular docking parameters for the interaction of target protein, acetylcholinesterase with F0850-4777 and their respective control ligands.

Donor-Acceptor pair	Distance (Å)	Nature of Interaction	Docking energy, kcal mol^{-1}	Binding affinity, M^{-1}
AChE-Tacrine complex				
LIG:HN - ARG289:O	2.2889	Conventional Hydrogen Bond	-8.5	1.72×10^{6}
TYR121 - LIG	5.6263	Hydrophobic (Pi-Pi Stacked)		
TYR121 - LIG	4.1778	Hydrophobic (Pi-Pi Stacked)		
TRP279 - LIG	4.4472	Hydrophobic (Pi-Pi T-shaped)		
TYR121 - LIG	3.8452	Hydrophobic (Pi-Alkyl)		
TRP279 - LIG	4.6750	Hydrophobic (Pi-Alkyl)		
AChE-F0850-4777 complex				
LIG:C - PHE330	3.4976	Hydrophobic (Pi-Sigma)	-12.2	8.87×10^{8}
TRP84 - LIG	4.5801	Hydrophobic (Pi-Pi Stacked)		
TRP84 - LIG	4.2239	Hydrophobic (Pi-Pi Stacked)		
TYR121 - LIG	3.8900	Hydrophobic (Pi-Pi Stacked)		
TYR121 - LIG	5.5085	Hydrophobic (Pi-Pi T-shaped)		
PHE330 - LIG	4.6986	Hydrophobic (Pi-Pi T-shaped)		
PHE330 - LIG	5.3491	Hydrophobic (Pi-Pi T-shaped)		
TYR334 - LIG	5.9813	Hydrophobic (Pi-Pi T-shaped)		
TYR334 - LIG	5.1634	Hydrophobic (Pi-Pi T-shaped)		
TYR121 - LIG:C	3.9472	Hydrophobic (Pi-Alkyl)		
TRP279 - N:LIG:C	4.8095	Hydrophobic (Pi-Alkyl)		

Table S2: Molecular docking parameters for the interaction of target protein, butyrylcholinesterase with F0850-4777 and their respective control ligands.

Donor-Acceptor pair	Distance (Å)	Nature of Interaction	Docking energy, kcal mol^{-1}	Binding affinity, M^{-1}
BChE-Tacrine complex				
LIG:HN - HIS438:O	2.0571	Conventional Hydrogen Bond	-8.4	1.45×10^{6}
TRP82 - LIG	4.2341	Hydrophobic (Pi-Pi Stacked)		
TRP82 - LIG	3.6040	Hydrophobic (Pi-Pi Stacked)		
TRP82 - LIG	3.7794	Hydrophobic (Pi-Pi Stacked)		
TRP82 - LIG	4.2088	Hydrophobic (Pi-Pi Stacked)		
HIS438 - LIG	5.7005	Hydrophobic (Pi-Pi Stacked)		
HIS438 - LIG	5.8765	Hydrophobic (Pi-Pi Stacked)		
ALA328 - LIG	4.4453	Hydrophobic (Alkyl)		
TRP82 - LIG	4.6204	Hydrophobic (Pi-Alkyl)		
TRP430 - LIG	5.4972	Hydrophobic (Pi-Alkyl)		
BChE-F0850-4777 complex				
LIG:C - SER287:O	3.6398	Carbon Hydrogen Bond	-10.7	7.04×10^{7}
PRO285:CA - LIG	3.6622	Hydrophobic (Pi-Sigma)		
TRP82 - LIG	3.7792	Hydrophobic (Pi-Pi Stacked)		
TRP82 - LIG	3.9212	Hydrophobic (Pi-Pi Stacked)		
TYR332 - LIG	3.8850	Hydrophobic (Pi-Pi Stacked)		
TYR332 - LIG	4.0772	Hydrophobic (Pi-Pi Stacked)		
TRP82 - LIG:C	5.1275	Hydrophobic (Pi-Alkyl)		
TRP82 - LIG:C	4.1632	Hydrophobic (Pi-Alkyl)		

Table S3: Molecular docking parameters for the interaction of target protein, monoamine oxidase-A with F0850-4777 and their respective control ligands.

Donor-Acceptor pair	Distance (Å)	Nature of Interaction	Docking energy, kcal mol^{-1}	Binding affinity, M^{-1}
MAO-A-Harmine complex				
GLY67:CA - LIG:O	3.3663	Carbon Hydrogen Bond	-8.7	2.40×10^6
LIG:C - GLY443:O	3.7816	Carbon Hydrogen Bond		
TYR407 - LIG	4.7765	Hydrophobic (Pi-Pi Stacked)		
TYR407 - LIG	3.8339	Hydrophobic (Pi-Pi Stacked)		
TYR444 - LIG	4.8632	Hydrophobic (Pi-Pi Stacked)		
LIG - TYR407	3.9958	Hydrophobic (Pi-Pi Stacked)		
LIG - TYR444	5.7755	Hydrophobic (Pi-Pi Stacked)		
MAO-A-F0850-4777 complex				
TYR407:HH - LIG:O	2.2756	Conventional Hydrogen Bond	-13.6	9.44×10^9
LIG:C - TYR69:O	3.4247	Carbon Hydrogen Bond		
CYS323:SG - LIG	5.9588	Pi-Sulfur		
CYS406:SG - LIG	5.4859	Pi-Sulfur		
TYR407 - LIG	3.8042	Hydrophobic (Pi-Pi Stacked)		
TYR407 - LIG	3.9094	Hydrophobic (Pi-Pi Stacked)		
TYR444 - LIG	5.8240	Hydrophobic (Pi-Pi Stacked)		
LIG:C - VAL210	5.0456	Hydrophobic (Pi-Alkyl)		
LIG:C - CYS323	4.2301	Hydrophobic (Pi-Alkyl)		
LIG:C - ILE335	5.2395	Hydrophobic (Pi-Alkyl)		
LIG:C - LEU337	4.4962	Hydrophobic (Pi-Alkyl)		
LIG - MET445	5.4345	Hydrophobic (Pi-Alkyl)		
LIG - ILE335	4.2469	Hydrophobic (Pi-Alkyl)		
LIG - LEU337	5.2089	Hydrophobic (Pi-Alkyl)		

Table S4: Molecular docking parameters for the interaction of target protein, monoamine oxidase-B with F0850-4777 and their respective control ligands.

Donor-Acceptor pair	Distance (Å)	Nature of Interaction	Docking energy, kcal mol^{-1}	Binding affinity, M^{-1}
MAO-B-Safinamide complex				
LIG:H - LEU171:O	3.0603	Conventional Hydrogen Bond	-9.5	9.28×10^6
LIG:H - GLN206:OE1	2.7431	Conventional Hydrogen Bond		
LEU171:CD2 - LIG	3.7261	Hydrophobic (Pi-Sigma)		
LIG:C - TYR398	3.9522	Hydrophobic (Pi-Sigma)		
CYS172:SG - LIG	5.0033	Pi-Sulfur		
TYR326 - LIG	5.3392	Hydrophobic (Pi-Pi T-shaped)		
LIG - ILE199	4.6594	Hydrophobic (Pi-Alkyl)		
LIG - ILE316	4.8965	Hydrophobic (Pi-Alkyl)		
MAO-B-F0850-4777 complex				
TYR435:HH - LIG:O	2.8605	Conventional Hydrogen Bond	-12.5	1.47×10^9
LIG:C - TYR60:O	3.2181	Carbon Hydrogen Bond		
LIG:C - GLY434:O	3.3236	Carbon Hydrogen Bond		
LEU171:CD2 - LIG	3.5066	Hydrophobic (Pi-Sigma)		
ILE199:CA - LIG	3.9907	Hydrophobic (Pi-Sigma)		
CYS172:SG - LIG	5.1135	Pi-Sulfur		
CYS397:SG - LIG	5.9264	Pi-Sulfur		
MET436:SD - LIG	5.8185	Pi-Sulfur		
TYR398 - LIG	3.8319	Hydrophobic (Pi-Pi Stacked)		
TYR398 - LIG	3.8750	Hydrophobic (Pi-Pi Stacked)		
TYR326 - LIG	5.0666	Hydrophobic (Pi-Pi T-shaped)		
LIG:C - LEU171	5.0268	Hydrophobic (Alkyl)		
LIG:C - ILE199	4.1227	Hydrophobic (Alkyl)		
TYR326 - LIG:C	4.6830	Hydrophobic (Pi-Alkyl)		
